# When Monkeys Learn Directional Tasks, Neurons Learn Too

**DOI:** 10.1371/journal.pbio.0020060

**Published:** 2004-02-17

If you've ever hit a patch of ice on the road that sent your car swerving left while you resolutely—and futilely—steered right to get back in your lane, you've experienced what neuroscientists call a “visuomotor rotation task.” On a dry road, your response would have been appropriate. But under icy conditions, the same sensory cue produces a decidedly negative result: a car fishtailing out of control. While you're figuring out what movements will straighten out the car, the neurons in your primary motor cortex—the region of the brain responsible for movement—are taking notes. Chances are, your next icy encounter was less dramatic. But how does your brain learn to produce a different movement in response to the same visual cue?

Neuroscientists investigate such questions by recording and analyzing the electrical activity of neurons during learning and performance of new sensory-motor transformations. Such studies, for example, show that populations of neurons in different brain areas map sensory cues and desired arm motion by creating an internal representation of the corresponding sensory and motor coordinates in a way that allows flexible responses to changing conditions. In previous studies, Rony Paz and Eilon Vaadia, of The Hebrew University in Israel, found that neurons in the primary motor cortex that fire before monkeys move their arm in a particular direction have higher firing rates after the monkey learns to dissociate the arm direction from the cursor direction (an indicator of visual feedback). Interestingly, changes in activity preferentially occurred in a subset of neurons that were already tuned (that is, maximally activated during movement) to the direction experienced while learning.

While many studies indicate that learning new tasks can generate specific changes in brain activity, it had not been clear how or if such changes improve the internal representation inside the brain. Specifically, is the neuronal code any “better” after learning? Now Paz and Vaadia show that while these neurons are firing at higher rates they are also transmitting more information about specific task parameters.

Paz and Vaadia trained two rhesus monkeys to learn various visual-motor tasks—which involved operating a joystick to move a cursor on a screen—and then changed the relationship between the visual feedback (the cursor) and hand movement. Using information-theory analysis—which measures the amount of information that single neurons can tell about the movement—they were able to correlate neuron activity with direction of movement and, conversely, distinguish differences between directions based on neuron activity. Their analysis revealed that the neurons transmit more information about the direction of movement after the monkeys learn a task. To figure out what aspect of neuron activity conveys this improved information, Paz and Vaadia examined two features of neuron signaling—response variability and directional sensitivity—which they reasoned might plausibly accomplish this. Increased information content after learning a task, they found, corresponded to sensitivity to a single direction, and neurons attuned to that direction contributed to the increase.

These findings suggest that subsets of directionally sensitive neurons increase their firing rates to more finely tune their sensitivity to that direction. By successfully reconstructing the movement direction from the neuron signals captured after learning a task, Paz and Vaadia also demonstrate that the observed learning improvement can be extracted to predict behavior. The authors argue that their results suggest a close association between properties of neurons—such as directional tuning of cells—and learning a skill that is focused on the same parameter—in this case, direction. Together with results from visual and auditory areas, they propose that similar mechanisms may control the interplay between neurons and learning throughout the central nervous system.

**Figure:**
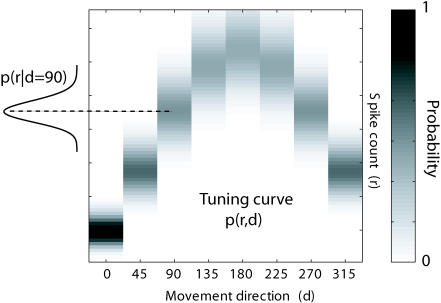
Mutual information between neuronal activity and direction of movement

